# Supplementary material for: Combined Tumor-Based BRCA1/2 and TP53 Mutation Testing in Ovarian Cancer
Source: Int J Mol Sci. 2023 Jul 18;24(14):11570. doi: 10.3390/ijms241411570 (PMC10380272; doi:10.3390/ijms241411570)
Supplement: Supplementary file 1 [file ijms-24-11570-s001.zip › ijms-2309018-supplementary.pdf]

Supplementary Table S1: *BRCA1/2* unclassified variants identified on tumor testing and their respective LOH analysis.

Patient	Gene	Variant	Protein	Functional domain	Impact	Allelic frequency	LOH	Germline variant	*TP53* associated mutation	Allelic frequency	Other associated variants
P2	BRCA2	c.8645A>C	p.Lys2882Thr	Tower	Missense	0,14	No	No	c.711G>A ;p.Met237Ile c.832 ; p.Pro278Ser	0,54	No
P3	BRCA2	c.6322C>T	p.Arg2108Cys	-	Missense	0,48	No	No	c.517G>A;p.Val173Met	0,59	No
P4	BRCA2	c.8351G>A	p.Arg2784Gln	OB1	Missense	0,86	Yes	No	c.527G>A;p.Cys176Tyr	0,89	UV ATR:c.7167T>G;p.Ile2389Met
P9	BRCA1	c.5096G>A	p.Arg1699Gln	BRCT1/AD2	Missense	0,79	Yes	Yes	c.722G>T;p.Glu258*	0,72	No
P12	BRCA1	c.287_292del	p.Asp96_Gly98de	Ring finger ° NES2	Small deletion	0,57	No	No	c.833_834insC; p.Gly279fs.	0,87	No
P15	BRCA2	c.6757C>G	p.Leu2253Val	-	Missense	0,15	No	No	c.702C>A; p.Tyr234*	0 ,68	UV ATM: c.4388T>G;p.Phe1463Cys UV ATM:c.6919C>T;p.Leu2307Phe
P20	BRCA1	c.1616C>T	p.Thr539Met	-	Missense	0,45	No	Yes	Non-contributory	Non-contributory	NR
P23	BRCA2	c.955A>T	p.Asn319Tyr	-	Missense	0 ,19	Yes	Yes	c.518T>G;p.Val173Gly	0 ,44	NR
P25	BRCA2	c.8435G>T	p.Gly2812Val	OB2	Missense	0,10	inconclusive	No	NR	NR	NR
P28	BRCA2	c.1096T>G	p.Leu366Val	-	Missense	0 ,18	Yes	No	c.202G>T ; p.Glu68*	0 ,45	NR
P31	BRCA2	c.122C>T	p.Pro41Leu	-	Missense	0,42	No	No	c.814G>A;p.Val272Met	0,66	No
P38	BRCA1	c.5165C>T	p.Ser1722Phe	BRCT1/AD2	Missense	0 ,41	Yes	Yes	c.782+1G>A	0,21	NF1:c.6854_6855insA,p.Tyr2285_Asn2286fs
P39	BRCA2	c.1607C>A	p.Ser536Tyr	-	Missense	0,50	inconclusive	Oui	No	WT	NR
P40	BRCA2	c.9586A>G	p.Lys3196Glu	-	Missense	0,45	No	Yes	c.309C>G ; p.Tyr103*	0,14	No

P43	BRCA 1	c.508C>T	p.Arg170Trp	-	Missense	0,16	Yes	Yes	c.645T>G ; p.Ser215Arg	0,70	No
P54	BRCA 1	c.300G>A	p.Glu100=	-	synonymous	0,08	inconclusive	inconclusive	c.584T>C;p.Ile195Thr	0,30	UV ATM:c.6068G>T;p.Gly2023Val<br>UV NF1:c.4749A>G;p.Glu1583Glu
P55	BRCA 2	c.7534C>T	p.Leu2512Phe	Helical domain	Missense	0,46	No	Yes	c.657_668del;p.Pro219_Pro223del	0,32	No
P56	BRCA 2	c.7791A>T	p.Lys2597Asn	Helical domain	Missense	0,03	Yes	NR	c.464C>A;p.Thr155Asn	0,0211	No
P59	BRCA 2	c.1275A>G	p.Glu425=	-	Missense	0,57	inconclusive	NR	c.670G>T; p.Glu224*	0,87	UV ATM:c.4258C>T; p.Leu1420Phe
P11	BRCA 2	c.856T>C	p.Ser286Pro	-	Missense	0,3	Yes	No	c.351delG; p.Gly117fs	0,29	BRCA1:c.5266_5267insC; p.Gln1756_Asp1757fs
P61	BRCA 2	c.6317T>C	p.Leu2106Pro	-	Missense	0 ,49	inconclusive	NR	c.856_861del; p.Glu286_Glu287del	0,19	UV ATM:c.1118C>G;p.Thr373Arg
P62	BRCA 2	c.4928T>C	p.Val1643Ala	-	Missense	0,40	Yes	Yes	c.224delC;p.Pro75fs	Not reported	
P50	BRCA 2	c.2133C>T	p.Cys711=	-		0,72	Yes	No	c.403T>G; p.Cys135Gly	0,62	UV NF1:c.369C>G;p.Thr123Thr<br>BRCA1:c.4072G>T ; p.Glu1358*
P47	BRCA 2	c.10121C>T	p.Thr3374Ile	-	Missense	0,71	inconclusive	NR	c.1010G>T ; p.Arg337Leu	0,52	BRCA1 c.3008_3009del
P64	BRCA 2	c.7719A>G	p.Leu2573=	Helical domain		0 ,57	inconclusive	inconclusive		No	ATM:c.609C>T,p.Asp203Asp

NR : not reported

Supplementary Table S2: List of *TP53* pathogenic mutations identified on tumor testing and their corresponding allelic frequency.

Patient	*TP53* mutation	Variant localization	Variant type	Variant frequency	Immunohistochemistry
P1	c.743 G>A;p.Arg248Gln	Exon 7	Missense	0,8	NR
P2	c.657_668del;p.Pro219_Pro223del	Exon 6	Frameshift	0,32	Positive
P3	c.375+1G>T; p.?	Intron 4	Splicing	0,45	NR
P4	c.97-1G>C ; p.?	Intron 3	Splicing	0,15	NR
P5	c.468_469insCCGGCACCCGC;p.Arg156_Val157fs	Exon 5	Frameshift	0,32	Negative
P6	c.711G>A p.Met237Ile	Exon 7	Missense	0,54	Positive
P7	c.517G>A;p.Val173Met	Exon 5	Missense	0,59	Positive
P8	c.672+1G>A; p.?	Intron 6	Splicing	0,15	Negative
P9	c.527G>A;p.Cys176Tyr	Exon 5	Missense	0,89	Positive
P10	c.976G>T;p.Glu326*	Exon 9	Nonsense	0,73	Positive
P11	c.394A>G ; p.Lys132Glu	Exon 5	Missense	0,63	NR
P12	c.809T>C; p.Phe270Ser	Exon 8	Missense	0,53	NR

P13	c.673-1G>C; p.?	Intron 6	Splicing	0,55	Negative
P14	c.464C>A;p.Thr155Asn	Exon 5	Missense	0,0211	NR
P15	c.307_308insGAAAACCT;p.Tyr103_Gln104fs	Exon 4	Frameshift	0,32	Negative
P16	c.262delG; p.Ala88fs	Exon 4	Frameshift	0,68	Negative
P17	c.742C>T;p.Arg248Trp	Exon 7	Missense	0,57	Positive
P18	c.587G>C;p.Arg196Pro	Exon 6	Missense	0,68	Positive
P19	c.764T>G; p.Ile255Thr	Exon 7	Missense	0,48	NR
P20	c.120_126del; p.Met40fs	Exon 4	Frameshift	0,59	Negative
P21	c.742C>T; p.Arg248Trp	Exon 7	Missense	0,91	Positive
P22	c.722G>T;p.Glu258*	Exon 7	Nonsense	0,72	Positive
P23	c.638G>T;p.Arg213Leu	Exon 6	Missense	0,52	NR
P24	c.406C>T;p.Gln136*	Exon 5	Nonsense	0,39	NR
P25	c.524 G>A;p.Arg175His	Exon 5	Missense	0,66	Positive
P26	c.743 G>A;p.Arg248Gln	Exon 7	Missense	0,64	Positive
P27	c.338T>C;p.Phe113Ser	Exon 4	Missense	0,32	Positive
P28	c.257_279del;p.Ala86fs	Exon 4	Frameshift	0,52	NR
P29	c.455_456insC; p.Pro152_Pro153fs	Exon 5	Frameshift	0,47	Negative
P30	c.670G>T; p.Glu224*	Exon 6	Nonsense	0,87	NR
P31	c.524 G>A;p.Arg175His	Exon 5	Missense	0,92	Positive
P32	c.578A>T;p.His193Leu	Exon 6	Missense	0,24	NR
P33	c.559+1G>T ; p.?	Intron 5	Splicing	0,1	Negative
P34	c.863del;p.Asn288fs	Exon 8	Frameshift	0,42	Positive
P35	c.775_776insG;p.Asp259_Ser260fs	Exon 7	Frameshift	0,8	NR
P36	c.351del; p.Gly117fs	Exon 4	Frameshift	0,29	NR
P37	c.701A>G;p.Tyr234Cys	Exon 7	Missense	0,7	NR
P38	c.464C>A,p.Thr155Asn	Exon 5	Missense	0,59	NR
P39	c.581T>C,p.Leu194Pro	Exon 6	Missense	0,59	Positive
P40	c.713G>A;p.Cys238Tyr	Exon 7	Missense	0,5	Positive
P41	c.393_395del;p.Asn131_Lys132del	Exon 5	Frameshift	0,6	NR
P42	c.833_834insC ; p.Gly279fs	Exon 8	Frameshift	Not reported	Negative
P43	c.542G>C;p.Arg181Pro	Exon 5	Missense	0,93	Positive
P44	c.766A>C;p.Thr256Pro	Exon 7	Missense	0,57	Positive
P45	c.569del;p.Pro190fs	Exon 6	Frameshift	O,60	NR

P46	c.856_861del;p.Glu286_Glu287del	Exon 8	Frameshift	0,19	Positive
P47	c.403T>C;p.Cys135Arg	Exon 5	Missense	0,18	Positive
P48	c.840A>C;p.Arg280Ser	Exon 8	Missense	0,62	NR
P49	c.702C>A;p.Tyr234*	Exon 7	Nonsense	0,68	Negative
P50	c.526T>A;p.Cys176Ser	Exon 5	Missense	Not reported	Positive
P51	c.613T>G;p.Tyr205Asp	Exon 6	Missense	0,38	NR
P52	c.646G>A;p.Val216Met	Exon 6	Missense	0,22	Positive
P53	c.375+5del;p.?	Intron 4	Splicing	0,057	Positive
P54	c.475G>C;p.Ala159Pro	Exon 5	Missense	0,07	Positive
P55	c.518T>C,p.Val173Ala	Exon 5	Missense	0,09	NR
P56	c.527G>A;p.Cys176Tyr	Exon 5	Missense	0,01	Positive
P57	c.672+1G>A;p.?	Intron 6	Splicing	0,75	Negative
P58	c.743G>A;p.Arg248Gln	Exon 7	Missense	0,61	Positive
P59	c.517G>T;p.Val173Leu	Exon 5	Missense	0,43	Positive
P60	c.422G>A;p.Cys141Tyr	Exon 5	Missense	0,29	NR
P61	c.224del;p.Pro75fs	Exon 4	Frameshift	Not reported	Negative
P62	c.743 G>A;p.Arg248Gln	Exon 7	Missense	0,22	NR
P63	c.1049T>C;p.Leu350Pro	Exon 10	Missense	0,48	NR
P64	c.517G>A;p.Val173Met	Exon 5	Missense	0,82	Positive
P65	c.832C>T;p.Pro278Ser	Exon 8	Missense	0,59	Positive
P66	c.713G>A,p.Cys238Tyr	Exon 7	Missense	0,71	NR
P67	c.527G>T;p.Cys176Phe	Exon 5	Missense	0,44	Positive
P68	c.518T>G;p.Val173Gly	Exon 5	Missense	0,44	Positive
P69	c.625A>T;p.Arg209*	Exon 6	Nonsense	0,61	Negative
P70	c.490A>G; p.Lys164Glu	Exon 5	Missense	0,35	Positive
P71	c.455_456insC;p.Pro152_Pro153fs	Exon 5	Frameshift	0,63	NR
P72	c.983del;p.Phe328fs	Exon 9	Frameshift	0,47	Negative
P73	c.824G>T,p.Cys275Phe	Exon 8	Missense	Not reported	Positive
P74	c.711G>A;p.Met237Ile	Exon 7	Missense	0,62	Positive
P75	c.146delA;p.Asp49fs	Exon 4	Frameshift	0,36	Positive
P76	c.742 C>T;p.Arg248Trp	Exon 7	Missense	0,40	Positive
P77	c.742C>T,p.Arg248Trp	Exon 7	Missense	0,15	NR
P78	c.470T>G;p.Val157Gly	Exon 5	Missense	0,35	Positive

P79	c.856G>T;p.Glu286*	Exon 8	Nonsense	0,78	NR
P80	c.916C>T ; p.Arg306*	Exon 8	Nonsense	0,48	Positive
P81	c.373A>C;p.Thr125Pro	Exon 4	Missense	0,74	NR
P82	c.395A>G;p.Lys132Arg	Exon 5	Missense	0,27	Positive
P83	c.818G>A,p.Arg273His	Exon 8	Missense	0,2	Positive
P84	c.488_489insTA;p.Lys164fs	Exon 5	Frameshift	0,79	NR
P85	c.150T>A;p.Ile50Ile p.Glu51Lysfs*67	Exon 4	Nonsense	0,14	Negative
P85	c.151_166delGAACAATGGTTCACTG;p.Glu51fs	Exon 4	Frameshift	0,14	Negative
P86	c.202G>T ; p.Glu68*	Exon 4	Nonsense	0,45	Negative
P87	c.1024C>T ; p.Arg342*	Exon 10	Nonsense	0,59	Negative
P88	c.584T>C;p.Ile195Thr	Exon 6	Missense	0,30	NR
P89	c.583A>T;p.Ile195Phe	Exon 6	Missense	0,07	NR
P90	c.402T>G;p.Phe134Leu	Exon 5	Missense	0,84	NR
P91	c.994-1G>A;p.?	Intron 9	Splicing	0,11	NR
P92	c.839G>C;p.Arg280Thr	Exon 8	Missense	0,47	Positive
P93	c.833C>T;p.Pro278Leu	Exon 8	Missense	0,76	Positive
P94	c.586C>T;p.Arg196*	Exon 6	Nonsense	0,67	Positive
P95	c.707A>G;p.Tyr236Cys	Exon 7	Missense	0,48	Negative
P96	c.542_559+6del;p.?	Exon 5	Splicing	0,26	Positive
P97	c.934dup ; p.Thr312fs	Exon 9	Frameshift	0,61	Negative
P98	c.711G>A ; p.Met237Ile	Exon 7	Missense	0,39	NR
P99	c.388C>T;p.Leu130Phe	Exon 5	Missense	0,02	NR
P100	c.524 G>A ; p.Arg175His	Exon 5	Missense	0,83	NR
P101	c.586C>T, p.Arg196*	Exon 6	Nonsense	0,20	Positive
P102	c.614A>G;p.Tyr205Cys	Exon 6	Missense	0,54	NR
P103	c.844C>T;p.Arg282Trp	Exon 8	Missense	0,81	Positive
P104	c.574C>T; p.Gln92*	Exon 6	Nonsense	0,85	Positive
P105	c.747G>T;p.Arg249Ser	Exon 7	Missense	0,04	NR
P106	c.681_682insT;p.Ser227_Asp228fs	Exon 7	Frameshift	0,42	Positive
P107	c.216_217insC;p.Pro72fs	Exon 4	Frameshift	0,69	Negative
P108	c.403T>G;p.Cys135Gly	Exon 5	Missense	0,62	Negative
P109	c.818_827del; p.Arg273fs	Exon 8	Frameshift	Not reported	NR
P110	c.450_473del;p.Thr150_Arg158del	Exon 5	Frameshift	0,53	Positive

P111	c.783-1_784delinsCTT;p.?	Intron 7	Splicing	0,20	NR
P112	c.818_827del;p.Arg273fs	Exon 8	Frameshift	0,25	Negative
P113	c.380C>T;p.Ser127Phe	Exon 5	Missense	0,62	NR
P114	c.814G>A;p.Val272Met	Exon 8	Missense	0,66	Positive
P115	c.824G>A;p.Cys275Tyr	Exon 8	Missense	0,62	NR
P116	c.524G>A;p.Arg175His	Exon 5	Missense	0,47	Positive
P117	c.644G>T,p.Ser215Ile	Exon 6	Missense	0,05	NR
P118	c.422G>A;p.Cys141Tyr	Exon 5	Missense	0,68	NR
P119	c.517G>T ; p.Val173Leu	Exon 5	Missense	0,02	Positive
P120	c.586C>T;p.ARG196*	Exon 6	Nonsense	0,26	NR
P121	c.659A>G;p.Tyr220Cys	Exon 6	Missense	0,22	NR
P122	c.151delG;p.Glu51fs	Exon 4	Frameshift	0,52	NR
P123	c.817 C>T; p.Arg273Cys	Exon 8	Missense	0,16	Negative
P124	c.394A>G;p.Lys132Glu	Exon 5	Missense	0,15	Positive
P125	c.159_198del,p.Trp53fs	Exon 4	Frameshift	0,15	NR
P126	c.782+1G>A	Intron 7	Splicing	0,21	NR
P127	c.742 C>T;p.Arg248Trp	Exon 7	Missense	0,27	NR
P128	c.461G>T;p.Gly154Val	Exon 5	Missense	0,71	NR
P129	c.526T>G ; p.Cys176Glu	Exon 5	Missense	0,72	Positive
P130	c.309C>G ; p.Tyr103*	Exon 4	Nonsense	0,14	Positive
P131	c.711G>T ; p.Met237Ile	Exon 7	Missense	0,75	Negative
P132	c.445_446insT ; p.Ser149fs	Exon 5	Frameshift	0,43	Positive
P133	c.610G>T ; p.Glu204*	Exon 6	Nonsense	0,93	NR
P134	c.974_984del ; p.Gly325fs	Exon 9	Frameshift	0,42	NR
P135	c.659A>G ; p.Tyr220Cys	Exon 6	Missense	0,76	NR
P136	c.1025G>A ; p.Arg342Gln	Exon 10	Missense	0,35	NR
P137	c.524 G>A ; p.Arg175His	Exon 5	Missense	0,42	NR
P138	c.392A>T ; p.Asn131Ile	Exon 5	Missense	0,46	NR
P139	c.439_449del ; p.p.Val147fs	Exon 5	Frameshift	0,52	NR
P140	c.645T>G ; p.Ser215Arg	Exon 6	Missense	0,70	Negative
P141	c.743 G>A ; p.Arg248Gln	Exon 7	Missense	0,80	Positive
P142	c.398T>G ; p.Met133Arg	Exon 5	Missense	0,35	Positive
P143	c.766_767insA ; p.Thr256_Leu257fs	Exon 7	Frameshift	0,54	Positive

P144	c.537T>A ; p.His179Gln	Exon 5	Missense	0,75	NR
P145	c.955_956del ; p.Lys319fs	Exon 9	Frameshift	0,40	Positive
P146	c.517G>T ; p.Val173Leu	Exon 5	Missense	0,43	Negative
P147	c.376-2A>G	Intron 4	Splicing	0,70	Positive
P148	c.706T>C ; p.Tyr236His	Exon 7	Missense	0,35	Positive
P149	c.818G>T ; p.Arg273Leu	Exon 8	Missense	0,72	NR
P150	c.824G>A ; p.Cys275Tyr	Exon 8	Missense	0,74	Positive
P151	c.824G>A ; p.Cys275Tyr	Exon 8	Missense	0,22	Positive
P152	c.396G>T ; p.Lys132Asn	Exon 5	Missense	0,64	Positive
P153	c.338T>C ; p.Phe113Ser	Exon 4	Missense	0,89	Positive
P154	c.713G>T ; p.Cys238Phe	Exon 7	Missense	0,33	NR
P155	c.1010G>T ; p.Arg337Leu	Exon 10	Missense	0,52	NR
P156	c.1024C>T ; p.Arg342*	Exon 10	Nonsense	0,19	Positive
P157	c.273G>A ; p.Trp91*	Exon 4	Nonsense	0,28	Positive
P158	c.734G>A ; p.Gly245Asp	Exon 7	Missense	0,39	NR
P159	c.659A>G ; p.Tyr220Cys	Exon 6	Missense	0,72	NR
P160	c.824G>A ; p.Cys275Tyr	Exon 8	Missense	0,62	Positive
P161	c.743 G>A ; p.Arg248Gln	Exon 7	Missense	0,53	Positive
P162	c.818 G>T ; p.Arg273Leu	Exon 8	Missense	0,48	NR
P163	c.376-2A>G	Intron 4	Splicing	0,90	Positive
P164	c.818 G>A ; p.Arg273His	Exon 8	Missense	0,56	NR
P165	c.637C>T ; p.Arg213*	Exon 6	Nonsense	0,71	NR
P166	c.209_231del ; p.Ala70fs	Exon 4	Frameshift	0,56	NR
P167	c.818G>T ; p.Arg273Leu	Exon 8	Missense	0,23	NR
P168	c.658T>C ; p.Tyr220His	Exon 6	Missense	0,60	NR

A/

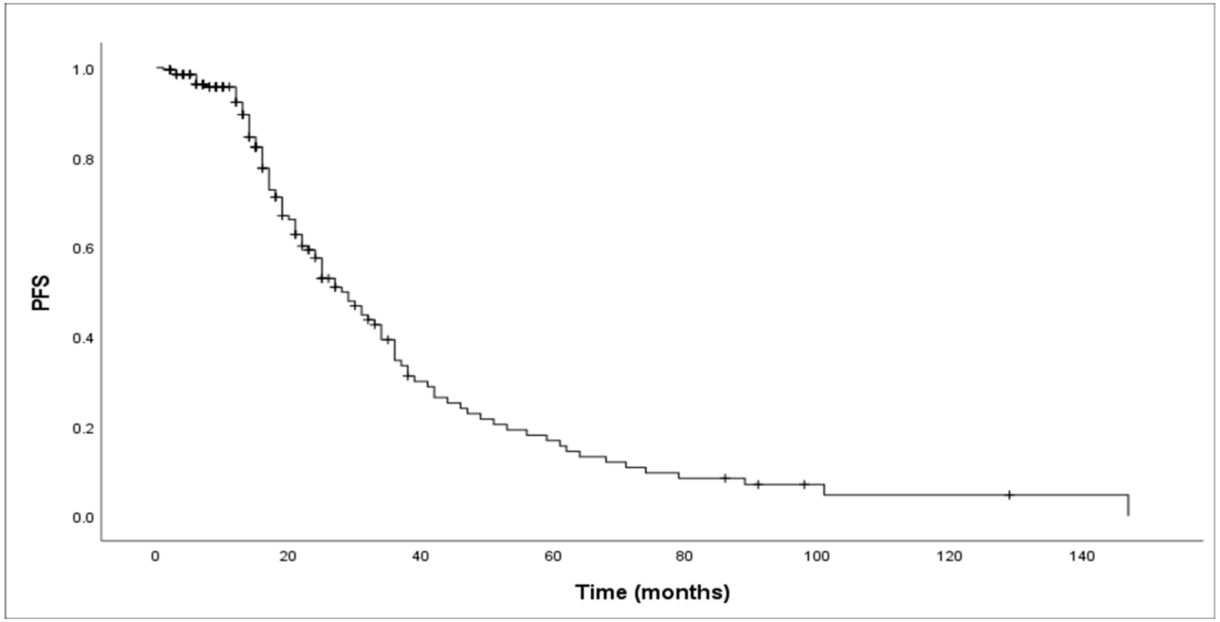

B/

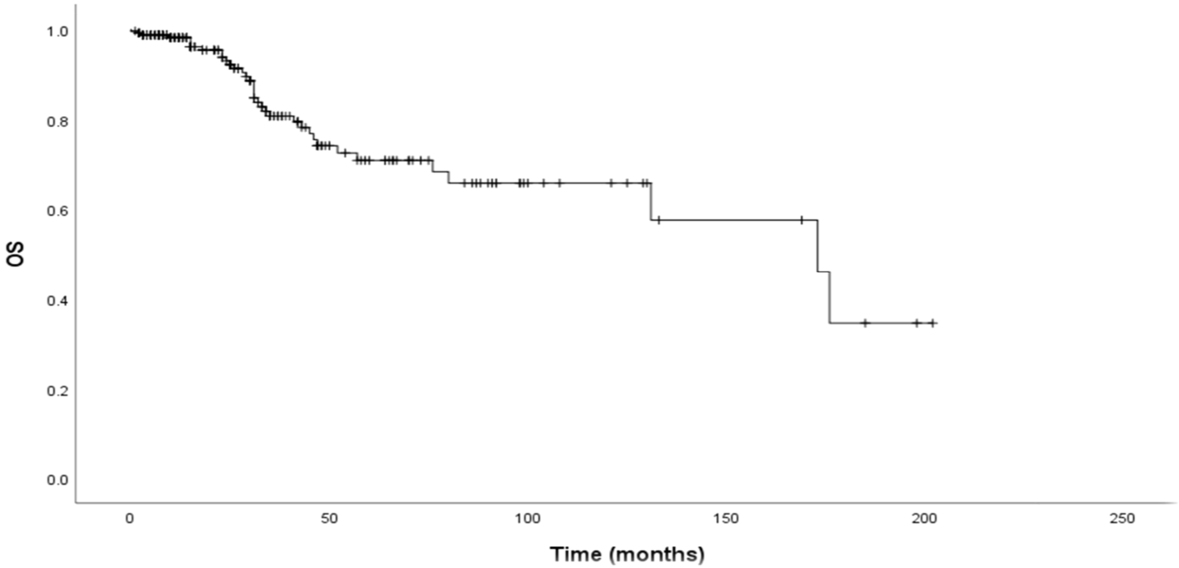

Supplementary Figure S1. A/ Progression free survival (PFS) curve oh the cohort. B/Overall survival (OS) curve of the cohort.
